# Supplementary material for: Genome sequencing is critical for forecasting outcomes following congenital cardiac surgery
Source: Nat Commun. 2025 Jul 10;16:6365. doi: 10.1038/s41467-025-61625-0 (PMC12246213; doi:10.1038/s41467-025-61625-0)
Supplement: Supplementary file 2 — Description of Additional Supplementary Files [file 41467_2025_61625_MOESM2_ESM.pdf]

Description of Additional Supplementary Files

Supplementary Data 1: A list of *de novo* and dominant damaging genetic variants and their attributes identified by GEM in 2,253 CHD patients.

Supplementary Data 2: Damaging biallelic variants and their attributes identified by GEM in 2,253 CHD patients.

Supplementary Data 3: Nine gene lists used for analysis. Lists were compiled from the literature and from pathways using reactome.org.

Supplementary Data 4: Conditional and final counts for Bayesian risk calculations shown in Figures 1c, 2, and 3.

Supplementary Data 5: A list of the ECAs and human phenotype ontology (HPO) terms for the 898 of the 2,253 CHD patients with reported ECAs.

Supplementary Data 6: A list of the rate of occurrence of ECAs in the CHD cohort, in CHD patients with damaging chromatin genotypes, in patients with damaging cilia-related genotypes, and in patients with reported mortality.

Supplementary Data 7: Fyler codes percentages observed in the CHD patients broken down by AVC, CTD, HTX, LVO, and OTH phenotype classes.

Supplementary Data 8: The binary phenotype matrix for 3,000 physician-classified CHD patients used to train the XGBoost classifier model. Column 2 shows the phenotype classes: 1 = AVC, 2 = CTD, 3 = HTX, 4 = LVO, and 5 = OTH.

Supplementary Data 9: Confusion matrix showing the correct and incorrect classification counts of all training samples for the final XGBoost model.

Supplementary Data 10: Standard multiclass summary statistics for classification of the training samples.

Supplementary Data 11: The XGBoost-based automated phenotype classifier predictions for 14,765 PCGC patients. Prediction probabilities for each phenotype class for each patient are shown. The prediction differential indicates the confidence (0 – 100) in the predicted assignment.

Supplementary Data 12: A list of variables and their frequencies used for Bayesian-based risk calculations.

Supplementary Data 13: A binary matrix of genetic and phenotypic variables in all CHD patients used to construct exact Bayesian networks and predict risk ratios.
