# Supplementary material for: Genome sequencing is critical for forecasting outcomes following congenital cardiac surgery
Source: Nat Commun. 2025 Jul 10;16:6365. doi: 10.1038/s41467-025-61625-0 (PMC12246213; doi:10.1038/s41467-025-61625-0)
Supplement: Supplementary file 4 — Reporting Summary [file 41467_2025_61625_MOESM4_ESM.pdf]

nature portfolio

Corresponding author(s): Dr. Martin Tristani-Firouzi

Last updated by author(s): May 24, 2024

Reporting Summary

Nature Portfolio wishes to improve the reproducibility of the work that we publish. This form provides structure for consistency and transparency in reporting. For further information on Nature Portfolio policies, see our Editorial Policies and the Editorial Policy Checklist.

Statistics

For all statistical analyses, confirm that the following items are present in the figure legend, table legend, main text, or Methods section.

n/a	Confirmed	
☐	☒	The exact sample size (*n*) for each experimental group/condition, given as a discrete number and unit of measurement
☐	☒	A statement on whether measurements were taken from distinct samples or whether the same sample was measured repeatedly
☐	☒	The statistical test(s) used AND whether they are one- or two-sided<br>*Only common tests should be described solely by name; describe more complex techniques in the Methods section.*
☐	☒	A description of all covariates tested
☐	☒	A description of any assumptions or corrections, such as tests of normality and adjustment for multiple comparisons
☐	☒	A full description of the statistical parameters including central tendency (e.g. means) or other basic estimates (e.g. regression coefficient) AND variation (e.g. standard deviation) or associated estimates of uncertainty (e.g. confidence intervals)
☐	☒	For null hypothesis testing, the test statistic (e.g. *F*, *t*, *r*) with confidence intervals, effect sizes, degrees of freedom and *P* value noted<br>*Give P values as exact values whenever suitable.*
☐	☒	For Bayesian analysis, information on the choice of priors and Markov chain Monte Carlo settings
☐	☒	For hierarchical and complex designs, identification of the appropriate level for tests and full reporting of outcomes
☐	☒	Estimates of effect sizes (e.g. Cohen's *d*, Pearson's *r*), indicating how they were calculated

Our web collection on statistics for biologists contains articles on many of the points above.

Software and code

Policy information about availability of computer code

Data collection	Patient data were collected from multiple medical centers participating in the Cardiac Genomics Consortium and Bench to Bassinet research programs [https://heartsmart.pcgcid.org/]. Surgical data, collected from multiple sites, were compiled by the Society of Thoracic Surgeons [https://www.sts.org/registries/sts-national-database]. Exome sequencing data have been deposited in the database of Genotypes and Phenotypes (dbGaP) under accession numbers phs000571.v1.p1, phs000571.v2.p1 and phs000571.v3.p2. Genetic and phenotypic data used in the paper are provided in the supplementary tables.
Data analysis	Phenotype classification was performed using the XGBoost software package [https://xgboost.ai/; https://github.com/dmlc/XGBoost.jl]. Bayesian network analyses were performed in R [https://www.r-project.org/] and Julia [https://julialang.org/] using the bnstruct R library and Julia code [https://github.com/ScottWatkins/BayesNetExplorer]. Gene pathway analysis was performed using the tools available at reactome [https://reactome.org]. Damaging variants were identified by GEM, which can be licensed through Fabric genomics [https://fabricgenomics.com/].

For manuscripts utilizing custom algorithms or software that are central to the research but not yet described in published literature, software must be made available to editors and reviewers. We strongly encourage code deposition in a community repository (e.g. GitHub). See the Nature Portfolio guidelines for submitting code & software for further information.

Data

Policy information about availability of data

All manuscripts must include a data availability statement. This statement should provide the following information, where applicable:

- Accession codes, unique identifiers, or web links for publicly available datasets
- A description of any restrictions on data availability
- For clinical datasets or third party data, please ensure that the statement adheres to our policy

Genetic and phenotypic data used in the paper are provided in the Supplementary Data file. Exome sequencing data have been deposited in the database of Genotypes and Phenotypes (dbGaP) under accession numbers phs000571.v1.p1, phs000571.v2.p1 and phs000571.v3.p2. Extended data for the CHD patients can be obtained through controlled access from the HeartsMart database [https://heartsmart.pcgcid.org/].

Research involving human participants, their data, or biological material

Policy information about studies with human participants or human data. See also policy information about sex, gender (identity/presentation), and sexual orientation and race, ethnicity and racism.

Reporting on sex and gender	Participants were 59% male and 41% female. Patients were not analyzed with respect to race, ethnicity, or gender.
Reporting on race, ethnicity, or other socially relevant groupings	NA
Population characteristics	Patients analyzed in the study are pediatric patients with severe congenital cardiac defects. All patients underwent one or more cardiac surgeries and were followed longitudinally throughout their participation in the Pediatric Cardiac Genomics Consortium (PCGC). All patients and patients data were de-identified with regards to this study.
Recruitment	Patients with congenital heart defects (CHDs) were recruited from multiple hospitals in the United States and England. Patient recruitment emphasized sporadic CHDs rather than familial or syndromic heart disease.
Ethics oversight	All patients were diagnosed, phenotyped, and recruited by PCGC centers and participating regional hospitals into the PCGC Congenital Heart Disease Network Study (CHD GENES: ClinicalTrials.gov identifier NCT01196182. Research at individual academic centers was performed under institutional review board (IRB) protocols specific to each center.

Note that full information on the approval of the study protocol must also be provided in the manuscript.

Field-specific reporting

Please select the one below that is the best fit for your research. If you are not sure, read the appropriate sections before making your selection.

☒ Life sciences ☐ Behavioural & social sciences ☐ Ecological, evolutionary & environmental sciences

For a reference copy of the document with all sections, see nature.com/documents/nr-reporting-summary-flat.pdf

Life sciences study design

All studies must disclose on these points even when the disclosure is negative.

Sample size	We included all patients (2,253) for which exome sequence data, cardiac phenotype data, and surgical outcomes data was available.
Data exclusions	Patients were limited to those with ≤ 10% missing data for surgical outcomes.
Replication	We applied cross-validation and re-sampling procedures to address reproducibility and confidence intervals. This is the largest cohort of CHD patients available and, at this time, no other comparable cohort exists for independent replication.
Randomization	All reported credible intervals were produced by random re-sampling to produce empirical 95% confidence intervals.
Blinding	Investigators were not blinded to the patients' CHD phenotypes.

Reporting for specific materials, systems and methods

We require information from authors about some types of materials, experimental systems and methods used in many studies. Here, indicate whether each material, system or method listed is relevant to your study. If you are not sure if a list item applies to your research, read the appropriate section before selecting a response.

Materials & experimental systems

n/a	Involved in the study
☒	☐ Antibodies
☒	☐ Eukaryotic cell lines
☒	☐ Palaeontology and archaeology
☒	☐ Animals and other organisms
☐	☒ Clinical data
☒	☐ Dual use research of concern
☒	☐ Plants

Methods

n/a	Involved in the study
☒	☐ ChIP-seq
☒	☐ Flow cytometry
☒	☐ MRI-based neuroimaging

Clinical data

Policy information about clinical studies

All manuscripts should comply with the ICMJE guidelines for publication of clinical research and a completed CONSORT checklist must be included with all submissions.

Clinical trial registration: NCT01196182

Study protocol: https://clinicaltrials.gov

Data collection: Patient were recruited from 2009-present at the following major hospitals: Boston's Children's Hospital, Brigham and Women's Hospital, Great Ormond Street Hospital, Children's Hospital of Los Angeles, Children's Hospital of Philadelphia, Columbia University Medical Center, Icahn School of Medicine at Mount Sinai, Rochester School of Medicine and Dentistry, Steven and Alexandra Cohen Children's Medical Center of New York, Lucile Packard Children's Hospital Stanford, University of California-San Francisco, University of Utah, and Yale School of Medicine. Phenotype and surgical data were reported by these sites. Exome sequencing was performed at multiple sites using only Illumina sequencing technology.

Outcomes: Primary outcomes were obtained from the Society of Thoracic Surgeons database. We screened these primary outcomes for association with damaging genetic variants. Outcomes with a positive association that were potentially actionable at the clinical level were selected for relative risk analysis. Relative risk estimates were calculated using Bayesian network analysis.

Plants

Seed stocks: NA

Novel plant genotypes: NA

Authentication: NA
